# More than fear? Brain activation patterns of dental phobic patients before and after an exposure-based treatment

**DOI:** 10.1007/s00702-024-02754-6

**Published:** 2024-03-01

**Authors:** André Wannemueller, Jürgen Margraf, Martin Busch, Hans-Peter Jöhren, Boris Suchan

**Affiliations:** 1 Center for the Study and Treatment of Mental Health, Ruhr University Bochum, Massenbergstr. 9-13, 44787 Bochum, Germany; 2 Grönemeyer-Institut for Microtherapy, Bochum, Germany; 3 Dental Clinic Bochum, Bochum, Germany; 4 Clinical Neuropsychology, Neuropsychological Therapy Centre, Ruhr University, Bochum, Germany

**Keywords:** Dental phobia, Functional magnetic resonance imaging, Cognitive behavioural therapy, Precuneus, Lateral parietal cortex, Exposure treatment

## Abstract

Hyperactivation of brain networks conferring defensive mobilization is assumed to underlie inappropriate defensive-preparation in patients with Specific Phobia. However, studies targeting Dental Phobia (DP) yielded quite heterogeneous results and research concerning the effects of exposure treatments on phobic brain activation so far is missing. This functional Magnetic Resonance Imaging (fMRI) study aimed to investigate activation patterns in DP patients during exposure to phobia-related stimuli and the effects of an exposure-based fear treatment on phobia-related activation. Seventeen patients with DP and seventeen non-phobic, healthy controls participated in this fMRI experiment presenting dental-related and neutral auditory and visual stimuli. After completing a short exposure-based CBT program, patients were scanned a second time to illustrate treatment-related changes in brain activation patterns. Pre-treatment fMRI results demonstrate enhanced activation in DP-patients mainly in the precuneus and lateral parietal cortex. Moreover, a small activation focus was observed in the amygdala and anterior cingulate cortex (ACC) as parts of classically fear-related structures. Activation in all these clusters decreased significantly from pre- to post-treatment assessment and in the case of the ACC was correlated with dental fear reduction. Activation changes in the precuneus and lateral parietal cortex suggest a pronounced first-person perspective memory processing including a vivid recall of contextual information from an egocentric perspective triggered by exposure to phobia-related stimuli. Besides a treatment-sensitive hyperactivity of fear-sensitive structures, DP may also be characterized by a disturbed memory retrieval that can be reorganized by successful exposure treatment.

**Supplementary Information:**

The online version contains supplementary material available at 10.1007/s00702-024-02754-6.

## Introduction

Marked psychological and physiological fear responses during the presence or anticipation of the fear-eliciting stimulus are amongst the main features of phobic fears according to the DSM-5 (American Psychiatric Association 2015). Lab studies exposing phobic participants to phobia-related stimuli such as pictures have consistently demonstrated inappropriate physiological defensive preparation in individuals with phobic disorders. This includes exaggerated startle-reflex sensitivity (De Jong et al. 1991; Globisch et al. 1999; Hamm et al. 1997; Larsen et al. 2002; McTeague et al. 2009) and heart-rate acceleration (Globisch et al. 1999; Sartory et al. 1987) in response to phobic stimuli across a wide range of phobic fears, such as animal phobias, injection phobia and social phobia. Exaggerated defensive responding is thought to prepare behavioural mobilization and immediate flight-fight due to a hyper-responsive defensive system (Lang et al. 1997) conceptualized as the key psychopathological process underlying specific phobias (SPs) (Hamm and Weike 2005; McTeague et al. 2012).

Meta-analytical evidence from functional magnetic resonance imaging (fMRI) studies (Ipser et al. 2013; Peñate et al. 2017) suggests enhanced activation of structures which are also known to be involved in conditioned fear learning (Fullana et al. 2016; Shin and Liberzon 2010) reflecting inappropriate defensive-preparation in SPs. Across studies, increased activation of the left-hemispheric insula, amygdala, globus pallidus and thalamus during exposure to phobia-related stimuli are the most consistently reported findings. However, numerous studies (e.g. Paquette et al. 2003; Rauch et al. 1997) failed to find increased amygdala activation during exposure to phobia-related stimuli. As most studies investigated spider or small animal phobia, the universal validity and generalizability of findings to other SPs might be questionable.

Dental Phobia (DP) is considered a highly impairing SP associated with significant oral health issues (Ng and Leung 2008), altered life quality (Vermaire et al. 2008) and negative psychosocial consequences (Cohen et al. 2000). It is assigned to the blood-injury phobia subtype. However, rather atypical for the blood-injection-injury phobia subtype, in patients with DP a pattern of exaggerated defence preparation with increased heart rate and startle-reflex potentiation during exposure to highly-arousing phobia-related contents has been demonstrated consistently (Sartory et al. 2009; Wannemueller et al. 2015a, 2017).

In contrast to peripheral physiological findings, research on DP-related brain activation so far yielded quite heterogeneous results. To the best of our knowledge, only four studies exist so far which have investigated neural activation of DP patients during exposure to dental-related stimuli (Hilbert et al. 2014; Lueken et al. 2011, 2014; Schienle et al. 2013): in one study (Lueken et al. 2014) no differential brain activation in any comparison of DP-patients compared to healthy controls (HC) was reported. In contrast to this, a typical pattern of amygdala-, hippocampus- and midbrain activation to phobia-related stimuli in a cohort of snake-phobic individuals was observed. A second study (Lueken et al. 2011) likewise did not find any difference in neural responding between patients with DP and HCs. Rather, DP-patients displayed a circumscribed activation pattern of increased prefrontal (PFC) and orbitofrontal cortex (OFC) activation. A more recent study (Hilbert et al. 2014) suggests that auditory but not visual stimulation might play a crucial role concerning the release of dental-related fear symptoms as reflected by increased activation in the insula, the anterior cingulate cortex (ACC), the orbitofrontal cortex (OFC), and the thalamus during auditory stimulation in DP. However, there is also one study (Schienle et al. 2013) suggesting brain-activation patterns of individuals with DP and animal phobia are similar as both groups displayed an increase of activation in the OFC, amygdala, supplementary motor areas (SMAs) and ACC in response to phobia related compared to neutral pictures. In line with this finding, a region of interest (RoI) analysis in a study using near-infrared techniques (Köchel et al. 2011) showed enhanced oxyhaemoglobin levels in the SMAs in dental phobic patients during auditory symptom provocation.

In sum, the reported results suggest that neural activation patterns in DP may at least partially be distinct from those observed in animal-phobic individuals such that exposure to fear-eliciting stimuli might be less associated with an immediate activation of the neural fear circuitry. This however contradicts peripheral-psychophysiological findings demonstrating defensive preparation in individuals with DP during symptom provocation (Sartory et al. 2009; Wannemueller et al. 2015a, 2017). However, in the case of DP, it may also be more difficult to identify differences in neuronal activation between phobic and non-phobic individuals, as on a physiological level also HCs display marked signs of defensive activation in response to dental-related stimulation (Wannemueller et al. 2017).

Today, a large number of studies have investigated the neuronal mechanisms underlying the extinction of lab-learned fear responses, i.e. neural responding to stimuli that formerly signalled threat but no longer do so. The majority of these studies reported an activation increase in the ventromedial prefrontal cortex (vmPFC) during extinction retention suggested to exhibit an inhibitory influence on amygdala activity (see Choy et al. 2007; Wolitzky-Taylor et al. 2008 for reviews). CBT-based treatments especially when including exposure elements have been evidenced to reduce subjective and behavioural phobic symptoms very successfully (Quirk and Mueller 2008; Sotres-Bayon et al. 2006). However, it is not clear whether neuronal correlates of successful exposure treatments mirror the findings described for the extinction of conditioned fear responses in specific phobias. To our knowledge, there are only a few studies with a total *N* of less than 100 patients (Goossens et al. 2007; Hauner et al. 2012; Ng and Leung 2008; Schienle et al. 2007; Straube et al. 2006), all conducted in spider phobia that have investigated changes in neuronal activation patterns following CBT-based treatments. One (Ng and Leung 2008) demonstrated a decline of right dorsolateral prefrontal cortex activation in patients viewing a phobia-related film excerpt after CBT. Three studies (Goossens et al. 2007; Ng and Leung 2008; Schienle et al. 2007; Straube et al. 2006) reported a decrease of insula/amygdala hyperactivity following CBT, with one reporting an additional decrease of ACC activation (Straube et al. 2006) and the other demonstrating an increase of the priorly reduced medial OFC activity after treatment **(**Schienle et al. 2007). Another study (Hauner et al. 2012) reported increases in prefrontal activity in conjunction with decreases in activity of the amygdala as a main result thereby emphasizing the close proximity of neural substrates of exposure treatment to those reported for experimental fear-extinction learning. A study by Halsband and Wolf 2015 could show significantly reduced amygdala, ACC, insula, and hippocampus activation in patients with DP when being exposed to dental-related stimuli under hypnosis compared to being in an awake state. This is to our knowledge the only study that investigated possible changes in brain activation pattern following psychological treatment in DP.

With respect to findings of the actual literature, the current fMRI study aimed to investigate differences in activation patterns during exposure to phobia-related visual and auditory stimuli compared to neutral stimulation in a group of dental phobic individuals (*n* = 17) in contrast to age and gender-matched healthy control group (*n* = 17).

Additionally, we investigated the effect of a highly standardized exposure-based fear treatment on phobia-related brain activation, by testing whether the activation of structures displaying differential activation between DP-patients and HCs in pre-treatment comparisons change after treatment and whether changes relate to treatment outcome. We expected to find increased activation within structures belonging to the fear circuitry during phobic stimulation in DP patients prior to the treatment. This would reflect psychophysiological findings in DP demonstrating defensive preparation in response to phobic stimulation in DP. Post-treatment, we expected to find decreased activation in those fear-related brain structures that should correspond to dental fear reduction.

## Methods

### Participants

Seventeen patients diagnosed with dental phobia (9 female/8 male) and 17 non-phobic controls (10 female/7 male) participated in this study, see Table 1. All phobic individuals were recruited at the ‘Treatment Centre for Dental Fear’ at a local dental clinic in Bochum (Germany) where they sought psychological care due to high and impairing dental fear. Individuals were asked to participate in the study if they met the criteria for a Specific (Dental-) Phobia according to the standards of DSM-IV (American Psychiatric Association 2000), did not take any psychopharmacological drugs, and were not pregnant, pierced or tattooed. Diagnoses and medical exclusion criteria were confirmed using the short form of the German Mini-DIPS (Margraf 1994) as a semi-structured clinical interview. The following comorbid disorders were diagnosed in DP-patients: 3 Specific Phobias (in addition to DP), 1 Social Phobia, 3 Substance Withdrawals (excluding nicotine).

**Table 1:** Sociodemographic variables, dental fear, and stimulus ratings in dental phobic individuals and controls without a history of dental phobia and results of within-subject comparisons (pre-treatment vs. post-treatment) in phobic individuals and results of between-subject comparisons (DP-Pat vs. HC) either containing the pre-treatment or post-treatment scores of phobic individuals

	Patients with dental phobia (DP-Pat, n = 17)			Controls (HC, n = 17)	DP-Pat (pre vs. post)	DP-Pat (pre) vs. HC	DP-Pat (post) vs. HC
	Pre M ± SD	Post M ± SD	Pre- to post change (%) M ± SD	M ± SD	T	T	T
Age (year)	39.41 ± 10.31	–	–	32.82 ± 13.52	–	1.60	–
Sex (female/male)	9/8	–	–	10 / 7	–	.73^**a**^	–
Education (year)	15.07 ± 4.85	–	–	17.75 ± 4.16	–	1.66	–
Dental fear							
DAS	18.76 ± 1.52	12.12 ± 3.08	34.78 ± 17.97	7.12 ± 1.45	7.57***	22.83***	6.91***
HAF	48.38 ± 5.23	32.70 ± 9.32	33.30 ± 22.25	–	6.01***	–	–
Sound ratings (1–4)							
Dental	2.74 ± 0.61	2.05 ± 0.67	24.25 ± 21.17	1.58 ± 0.34	4.02**	6.62***	2.42*
Neutral	1.64 ± 0.75	1.39 ± 0.73	11.37 ± 21.52	1.17 ± 0.12	1.51	2.37*	1.06
Picture ratings (1–4)							
Dental	2.76 ± 0.54	2.01 ± 0.72	27.68 ± 19.07	1.43 ± 0.37	5.04***	8.04***	2.72*
Neutral	1.24 ± 0.61	1.04 ± 0.06	8.25 ± 17.09	1.03 ± 0.38	1.31	1.36	1.08

*DAS* Dental Anxiety Scale; *HAF* Hierarchischer Angstfragebogen

*p < 0.05

**p < 0.01

***p < 0.001

Control subjects were asked to participate if they were not dental phobic, i.e. did not report any symptoms of dental fear or avoidance of dental treatments, scored beyond the cut-off score for mild dental fear in the Dental Anxiety Scale, i.e. displaying a score ≤ 12 (scale description see below) and denied any form of psychological treatment or psychotropic drug taking. Since no clinical interview was conducted with the control subjects, no statement can be made about any existing mental disorders in this subsample besides dental phobia.

All participants gave written informed consent before functional imaging started. The local ethics committee of the psychological faculty of the Ruhr University Bochum approved the study (number 060).

### Psychological treatment

A stress inoculation training (Meichenbaum 2007) adapted for the use in DP patients (Sartory and Wannemüller 2010; Wannemüller et al. 2015b) was administered in five standardized sessions imparting cognitive and bodily coping strategies to apply them in various exposure exercises. Session 1 consisted of conducting the diagnostic interview and imparting psycho-educative information about the sense of fear and its evolutionary function. Patients’ cognitive and physiological symptoms in a dental surgery situation were gathered and the rationale of helpful thoughts, breathing techniques and applied relaxation as cognitive and bodily coping strategies was explained. In order to prepare for the later usage in the dental situation, patients were asked to train Progressive Muscle Relaxation (PMR) in home exercises via CD-instructions. In the second session, a three-step program for applied relaxation (Öst 1987) based on the PMR concept was introduced. The program aimed at teaching an individualized form of short relaxation to be trained at home and applied against bodily fear symptoms in highly stressful situations. Moreover, the patients elaborated coping-oriented thoughts for the dental situation with the help of the therapist. The third session started with a video exposure of a dental situation (filling of a carious tooth). During the scene, patients should concentrate on their cognitive and physiological fear responses. Afterward, patients were invited to undergo a noise exposure exercise consisting of different dental-burr noises (turbine burr and rose-head burr). During exposure, patients were asked to cope with eliciting fear by applying a deep abdominal breathing technique instructed by the psychotherapist. The fourth and fifth sessions both consisted of therapist-guided in sensu exposure exercises both composed of vividly descriptions of a dental surgery. In the fourth session, the use of newly learned bodily and cognitive coping strategies was prompted by the therapist. In the fifth session, patients should cope with upcoming fear responses during exposure. Before treatment ended, patients required relapse-prevention strategies and were encouraged to directly arrange a dental appointment. Against the background of the treatment components described and the rationale of stress inoculation training, active coping of anxiety symptoms and the associated increase in the experience of control is the postulated key mechanism of action of the applied treatment. However, since repeated exposure exercises were also carried out with the patients, it is likely that habituation and inhibitory learning processes may also have occurred during treatment.

### Experimental procedure

The fMRI experiment was conducted at a local Medical Centre in Bochum, Germany (Grönemeyer Institute for Microtherapy). The pre-treatment scan of patients was conducted in the interval between treatment session 1 and treatment session 2, i.e. after the diagnostics and psychoeducation session and prior to the experience of any exposure treatment elements. Post-treatment scan was conducted within two weeks after completing the treatment.

After a short briefing about the upcoming procedure, participants entered the scanner. An MRI-suitable box with four response buttons was positioned on the participants’ abdomen. Prior to running the experiment the sound intensity was individually adapted to be well audible for the participants during the scanning, i.e. intense but not unpleasant. Eighty stimuli were presented in completely random order and were balanced with respect to valence (40 dental-related, 40 neutral) and modality (40 pictures, 40 sounds). Images were presented on a white screen which could be seen by the subjects via a double mirror with a 90° curve radius which was positioned at the head coil. Participants were equipped with MRI-suitable headphones. Presentation of each stimulus was preceded by a fixation cross on the screen presented for 1 s and followed by a blank with a randomly varying length between 1 and 2 s. Each stimulus was presented for 3 s. The fixation cross emerged again for 0.2 s on the screen after stimulus presentation. Three questions were presented for 3 s each. *“Please rate how unpleasant the picture/sound you just have seen/heard has been?”; “Please rate how arousing the picture/sound you just have seen/heard has been?”; “Please rate how fear-evoking the picture/sound you just have seen/heard has been?”.* Participants gave their responses via pressing the respective button (1–4). Additionally, a high-resolution (1 × 1 × 1 mm) T1-weighted structural image of the head was acquired after the experiment. The whole procedure took about 45 min for each participant.

### Stimuli

The 20 neutral pictures all derived from the International Affective Picture System (IAPS) (Lang et al. 2001). Neutral sounds were picked from the International Affective Digitalized Sound System (IADS) (Bradley and Lang 1999). Dental-related pictures were partly taken from free web-sources and partly from the IAPS. Dental related sounds were self-recorded and consisted of 12 dental burr noises (6 turbine burrs; 5 rose-head burrs), three ultrasonic tartar removers, three sonic tartar removers, and three suction devices to remove saliva from the mouth, see Table S1 for an overview of the applied stimuli.

### Dental fear measures

In order to assess dental fear on different levels we applied several questionnaires. We used the German version of the Dental Anxiety Scale (DAS) (Corah 1969) and the German ‘Hierarchischer Angstfragebogen’ (HAF) (Johren 1999) to assess the subjective component of dental fear. In both questionnaires, participants are asked to rate the extent of subjective fear in four (DAS) or respectively eleven (HAF) dental-related situations on a five-item scale (1–5). The authors of both instruments report sufficient to good internal consistencies, ranging from Cronbachs’s α = 0.64 (DAS) to α = 0.80 (HAF). Scores ≥ 15 in the DAS are considered to indicate high dental fear and scores of 13 and 14 moderate dental fear (Corah et al. 1987). According to the author of the HAF, a score > 38 very sensitively indicates dental phobia.

### Image acquisition

A total of 440 T2*-weighted whole brain volumes were acquired using a 1.5 T Symphony scanner (Siemens, Germany). Each volume consisted of 25 slices of 3 mm with an inter-slice gap of 1 mm. The repetition time was 80 ms per slice with a flip angle of 90°. We further acquired a high-resolution T1-weighted image with a voxel size of 1 × 1 × 1 mm and a repetition time of 2110 ms. The echo time was 3.93 ms with a flip angle of 15°. T1-weighted images were used for neuroanatomical localization and co-registration of the functional data.

### Data analyses

Functional data were analyzed using the latest version of SPM 12 software package (https://www.fil.ion.ucl.ac.uk/spm/). Images were slice time corrected realigned and unwarped in a second step. In the next step, these data were segmented using the tissue probability maps provided by SPM12. Images were then co-registered to the warped mean image. In the last step, images were normalized and smoothed with a Gaussian kernel of 8 mm. All functional imaging data were fed into a first-level analysis including five regressors: visual neutral images, visual phobic images, auditory neutral stimuli, auditory phobic stimuli and rating interval. Phobic stimuli were contrasted with neutral stimuli of the same modality. These contrast images were fed into a second-level analysis using an ANOVA with the factors Modality (visual and auditory) and Group (patients and healthy controls). Main effects and interactions were analysed using non-directional f-contrasts. Data were thresholded at p < 0.05 using a false discovery rate (fdr) correction and a minimum of 8 contiguous voxel. The resulting activation clusters were defined as regions of interest (ROI) for a pre-post analysis of patients’ activation patterns. Signal changes of these ROIs were extracted using the marsbar region of interest toolbox 0.44 (http://marsbar.sourceforge.net/). Extracted signal changes were further analysed using the statistical software package SPSS (IBM SPSS 26) by applying an ANOVA with the factors Modality (visual and auditory) and Time (pre and post) to all ROIs separately.

## Results

### Sample characteristics, subjective dental fear, and stimulus ratings

Sample characteristics, clinical data, subjective stimulus ratings as well as the results of pre- to post-treatment comparisons and between-subject comparisons are presented in Table 1. As intended, patients with DP did not differ from controls in regard to age, sex or educational level and showed highly significantly larger scores in both instruments measuring dental fear. This still–however to a much less extent–was the case when comparing the post-treatment scores of phobic participants to that of controls. Nevertheless, within phobic participants we observed substantial improvements across all dental instruments from pre- to post-treatment dental fear (mean change = 32.38% ± 17.78%).

Concerning the stimulus ratings at pre-treatment assessment a 2 (Group: DP vs. HC) × 2 (Modality: auditory vs. visual) × 2 (Valence: phobia related vs. neutral) repeated measure ANOVA yielded highly significant main effects of Group, *F*(1,28) = 35.14, *p* < 0.001, η^2^ = 0.58 (DP patients > HC); Modality, *F*(1,28) = 5.60, *p* = 0.025, η^2^ = 0.17 (sounds > pictures) and Valence, *F*(1,28) = 129.51, *p* < 0.001, η^2^ = 0.82 (dental > neutral). The Group x Valence effect, *F*(1,30) = 36.17, *p* < 0.001, η^2^ = 0.56, was the only significant interaction effect, see Table 1 for post-hoc test results. Repeated measures ANOVA within DP-patients yielded a highly significant effect of Time, *F*(1,13) = 14.48, *p* = 0.002, η^2^ = 0.53 (pre > post) and Valence, *F*(1,15) = 71.92,* p* < 0.001, η^2^ = 0.85 (dental > neutral). No interaction effect was significant.

When comparing the post-treatment stimulus ratings of DP patients with that of HCs the Group x Valence effect no longer existed, *F*(1,31) = 2.86, *p* < 0.03, η^2^ = 0.13, see Table 1 for post-hoc test results.

### Pre-treatment comparisons of phobia-related neural activation (phob. > neutr.) between healthy controls and DP patients

**Fig. 1:**
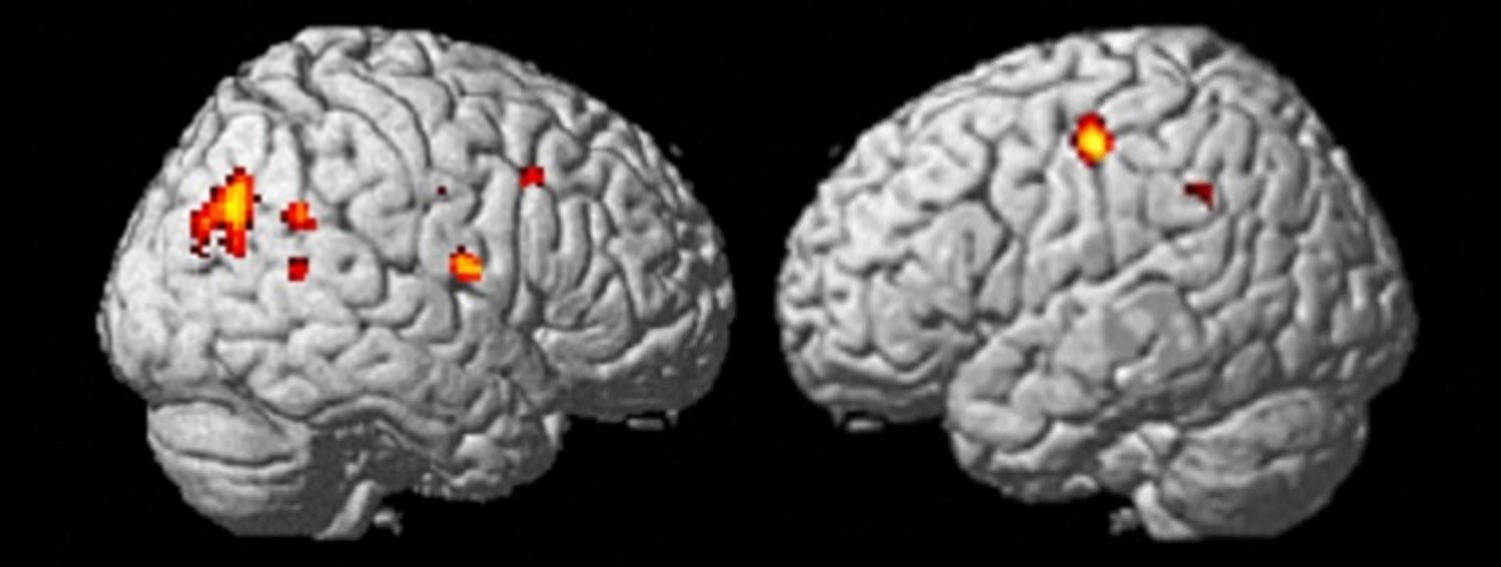
Significant activation clusters (p < 0.05, FDR-corrected) for the pre-treatment contrast DP > HC while processing phobic stimuli. Colours mark levels of F-score

No voxel survived the threshold for the interaction between Group and Modality. Only the main effect for Group yielded evidence for activation differences at the defined threshold of *p* < 0.05 (fdr corrected) in a big cluster covering the right precuneus and extending into the lateral parietal cortex covering Brodmann area (BA) 39 and BA 40 (see Table 2). Additionally, a cluster in the midline near precuneus (BA 7) was found. Further clusters were found in the left primary and right premotor cortex. The right supramarginal gyrus was also activated in this main effect. Further activation foci were found in the right insula, the anterior cingulate cortex (ACC) as well as in the superior temporal gyrus, see Table 2 and Fig. 1.

**Table 2:** Significant activation cluster (p < 0.05, FDR-corrected, with a minimum cluster size of 8 contiguous voxel) for the pre-treatment contrast DP > HC while processing phobic stimuli

Cluster size	x	y	z	Anatomical structure	BA
252	42	− 68	36	Precuneus	39
	44	− 68	24	Lateral parietal cortex	39
	42	− 78	26	Lateral parietal cortex	39
52	58	− 4	14	Precentral gyrus	6
50	46	− 50	30	Supramarginal gyrus	40
135	− 48	− 16	50	Precentral gyrus	4
	− 40	− 18	50	Precentral gyrus	4
40	40	− 20	− 10	Insula	13
48	30	− 20	38		
44	30	16	40	Middle frontal gyrus	8
	28	12	28		
137	− 2	− 46	50	Precuneus	7
	− 4	− 38	48	Precuneus	7
88	− 6	46	8	Anterior cingulate cortex	32
18	54	− 52	14	Superior temporal gyrus	22
22	40	− 10	36	Precentral gyrus	6
	32	− 12	32		
26	2	− 66	32	Precuneus	7
16	− 58	− 48	36	Supramarginal gyrus	40
8	− 12	− 28	60	Medial frontal gyrus	6

Data are presented in the space of the Montreal Neurological Institute (MNI) as provided by SPM. Probable Brodmann areas (BA) are also included

### Post-treatment activation changes in DP-patients

**Fig. 2:**
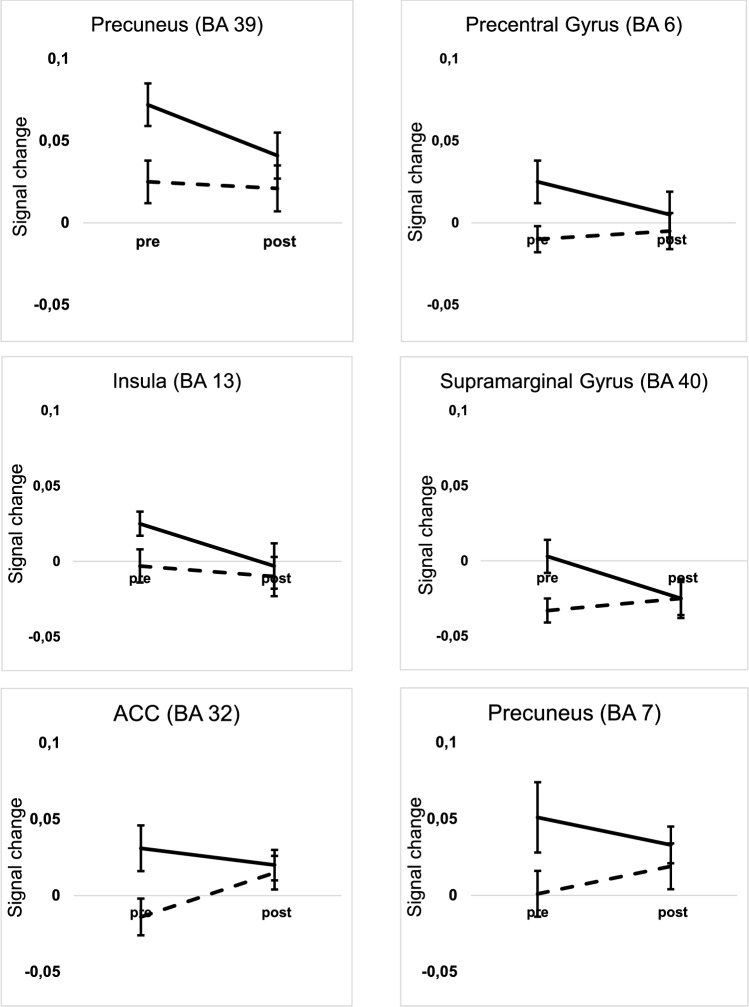
Course of signal changes from pre- to post-treatment in DP-patients in all six regions where DP-patients showed significantly larger differential brain activation than controls at pre-treatment assessment (phobia-related material solid line; neutral material dashed line)

The former described contrast illustrates the general activation difference between healthy controls and DPs during processing of visual and auditory phobic stimuli. To investigate pre-post related treatment changes and taking the general differences between healthy controls and DPs into account, activation clusters from the former described main effect (Group: healthy controls vs DP-patients pre-treatment) were used as ROIs for further analysis. Signal changes were extracted from these ROIs for pre- and post-treatment activation and fed into a repeated measures ANOVA including the factors Modality, Valence and Time (pre and post-treatment) in search for Valence × Time effects or Modality × Valence × Time effects. Results from all these analyses yielded evidence for significant Valence × Time effects in all extracted ROIs. Again, in no case any interaction effects containing the factor Modality or triple interaction effects reached the level of significance, see Fig. 2 for the course of signal changes in the respective ROIs.

### Associations between activation changes and clinical data

**Fig. 3:**
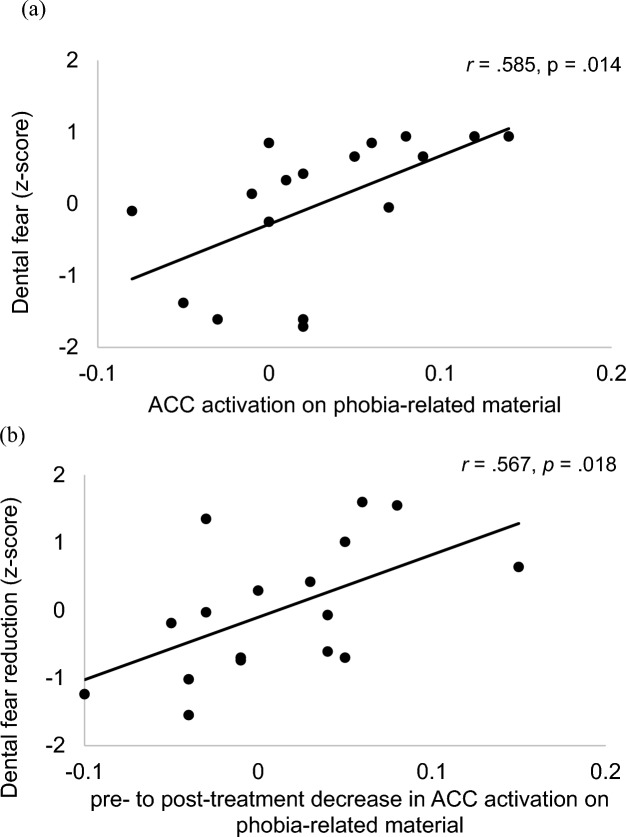
**a** Scatter plot of the correlation between pre-treatment ACC activation on phobia-related material and dental fear in patients with dental phobia. **b** Scatter plot of the correlation between post-treatment dental fear reduction and activation decrease in the ACC on phobia-related stimuli in patients with dental phobia

Correlation analyses examining associations between dental fear and signal changes at pre-treatment assessment in the extracted ROIs yielded a significant correlation between the signal change to phobia-related material in the ACC (BA 32) and pre-treatment subjective dental fear (see Fig. 3a).

Moreover, pre- to post-treatment activation decrease in this area was correlated with changes in dental fear after treatment (see Fig. 3b).

## Discussion

This study aimed to identify characteristic neural activation patterns in patients with DP during exposure to visual and auditory phobia-related stimuli. In addition, we investigated changes in brain activation patterns in DP patients after an exposure-based brief CBT in structures that previously differed from healthy controls.

As expected, there were large pre-treatment differences regarding dental fear levels as well as unpleasantness ratings of the applied dental-related stimuli between DP patients and HCs. Consistent with these subjective findings, an increase in activation was found in two clusters covering the right insula cortex (BA 13) and the ACC (BA 32) in patients during exposure to phobia-related stimuli. As studies investigating conditioned fear (Fullana et al. 2016; Shin and Liberzon 2010) and those focussing phobic fear responses (Goossens et al. 2007; Shin and Liberzon 2010) demonstrated hyperactivation in these structures during exposure to fear-related stimuli, both are counted among the key structures of the cerebral fear network (Shin and Liberzon 2010).

Concerning their respective role in acquiring and maintaining phobic fear responses lesion and pharmacological inhibition studies in rats suggest that the insula plays an important role in the consolidation of learned fear responses as well as in the learning of safety cues, which inhibit the expression of conditioned fear (Gogolla 2017), whereas medial prefrontal cortical regions including the ACC have been demonstrated to be critically involved in the expression of learned fear responses but were less important concerning the acquisition of fear learning itself. Interestingly, activation of the ACC was shown to play a special role in the recall of rather old, or remote compared to recent fear memories (see Dixaut and Gräff (2021) and Jacobs and Moghaddam (2021) for reviews). The latter at least to some extent may correspond to anecdotal reports of some DP patients who reported that during the experiment they felt ‘transported back’ to the dental treatment situation in which they acquired their dental fear.

In accordance with previous findings (Goossens et al. 2007; Straube et al. 2006), ACC and insula activation during exposure to phobia-related stimuli decreased after the exposure treatment. Moreover, at least in case of the ACC, decreasing activation was associated with subjective dental fear reduction following treatment (see Fig. 2b). In line with Amodio and Frith's functional classification of the medial frontal cortex (MFC) (2006), the current cluster within the ACC lies in the anterior rostral MFC region which is mainly activated in tasks requiring self-knowledge, such as the evaluation of self-related traits (Schmitz et al. 2004) and judgements of one’s own affective response (Ochsner et al. 2004; Zysset et al. 2002). This finding underlines the involvement of fear-sensitive structures in phobic dental anxiety and provides evidence for a neural substrate of successful fear reduction following exposure treatments may consist in down-regulating hyperactivity of these structures. Given the ACC findings, one could hypothesize that one consequence of successful treatments might be a lessening fixation on the assessment of one's emotional state during exposure to fear cues. The neurological correlate of this decreasing “state orientation” (Kuhl 1981) could thus be found in a decrease of activation in the anterior rostral MFC. Overall, the signs of defensive activation observed here on a central level, as well as the correspondingly reported peripheral physiological correlates, consisting of heart rate acceleration and startle potentiation reported in patients with DP (e.g. Wannemüller et al. 2017), rather suggest a special position of dental phobia in the subtype of blood injection injury phobia, where less sympathetically mediated, sometimes even diphasic fear responses are common.

Interestingly, with one exception (Schienle et al. 2013), studies focussing on DP so far had all major problems replicating the findings from fear conditioning studies and those conducted with animal-phobic individuals (Hilbert et al. 2014; Lueken et al. 2011, 2014). One possible reason for this could be that dental-related stimuli are generally unpleasant and trigger defensive activation per se in non-phobic participants as well (Wannemueller et al. 2017). This could make it harder to identify differential activation in the fear circuitry in DP, especially if a very sharp diagnostic line is not drawn between subclinically anxious and phobic patients. In many of the previous studies, the phobic sample was recruited from student samples with strikingly high questionnaire scores. However, our sample consisted of individuals who sought help in a specialised outpatient clinic because of their DP symptoms and avoidance of dental treatments. A general extensive assessment of dental fear also in healthy controls should be included in future studies. Another reason for partially inconsistent findings in DP and other SPs (as mentioned, most research so far refers to animal phobias, in particular spider phobia) may consist of factors such as pain perceiving or disgust may play different roles and could therefore also lead to differential patterns of neuronal activation during exposure to phobia-relevant stimuli. Moreover, it cannot be ruled out that the inconsistencies at least partially were simply due to artifacts caused by the overall small sample size of most fMRI studies.

Besides hyperactivity in fear-sensitive structures, results from the present study suggest that stimulus-driven memory and attention processes may crucially differ between DP-patients and HCs mainly reflected by higher activations of the right inferior parietal lobe (BA 39) and midline near precuneus (BA 7) in DP patients. BA 39 is thought to be incorporated in a fronto-parietal attention network which is especially involved in memory-guided attention and attention to memories as suggested in a recent meta-analysis (Fischer et al. 2021). Moreover, lesion studies, as well as results from fMRI studies, yield evidence for the importance of this parietal structure regarding the vividness of episodic memory content retrieval (see Rugg and King 2017; Sestieri et al. 2017 for recent reviews]. For example, patients with lesions in this area had deficits with spontaneous retrieval or free recall of spatial, emotional, perceptual and referential context information compared to control subjects, but were comparably good at providing information about those details when explicitly asked to (Berryhill et al. 2007; Davidson et al. 2008). In addition, lesions in this area did not lead to poorer overall performance in a cued recall test. However, patients were significantly more likely than controls to report that their knowledge was based on a more intuitive feeling of “familiarity”, i.e. knowing the stimulus without awareness of contextual information in which it has been encountered rather than “recollection”, defined as a vivid, clear “remembering” of an item and its surrounding contextual details (Tulving 1985). Other studies underline the importance of parietal functioning for recollecting details from an egocentric first-person perspective (Rorden et al. 2012; Russell et al. 2019) giving further insight in processing phobia related stimuli in DP.

Besides inferior parts of the right parietal lobe, the midline near precuneus (BA 7) was hyperactivated in DP patients during exposure to dental-related stimuli at pre-treatment assessment. In pioneering work by Fletcher and colleagues (Fletcher et al. 1995), this brain structure was named the “minds eye” as the authors could show that the precuneus was crucially involved in memory-related imagery processes. It has been demonstrated that the precuneus is also involved in a network related to the processing of contextual association and strongly associated to the Default Mode Network (DMN) (Raichle 2015) generally accepted as the principal brain locus of internal processing and self-generated cognition (e.g. Andrews-Hanna et al. 2014; Axelrod et al. 2017). Among other processes, the DMN is implicated in constructing (e.g. Addis et al. 2007) and retrieving episodic memories (e.g. Rugg and Vilberg 2013). This significant precuneus activation might therefore reflect strong imagery processes associated with phobia-related stimuli and illustrate the internal states of DPs during these processes.

The current findings in the inferior parietal lobe and midline near the precuneus region shed light on processing that may be even more benchmarking for DP than the above presented pattern found in the ACC and insula. They suggest a pronounced first-person perspective memory processing including a vivid recall of contextual information from an egocentric perspective in DP-patients when being exposed to phobia-related stimuli. This matches the unrecorded comments of many of the participating patients who reported feeling like “being at the dentist” again by seeing or hearing the dental-related stimuli. Furthermore, it matches study findings demonstrating that patients with DP show a strong tendency towards involuntarily retrieving severely disturbing imagery or mental recollections of former dental experiences (De Jongh et al. 2002). Among eleven evaluated situational fears DP by far was most strongly associated with intrusive re-experiencing of phobia-related events (Oosterink et al. 2009). Taken together, these findings suggest that patients with DP might have developed a pathological memory network in which emotional, sensory, perceptual, and cognitive elements related to dental treatment are stored and are extremely easily retrieved, as also suggested for Posttraumatic Stress Disorder (PTSD) (Brewin 2011). Indeed, nearly half of dental fearful patients have been shown to suffer from at least one PTSD symptom cluster, which in the majority of cases originated from dental-related experiences (De Jongh et al. 2003, 2006).

Post-treatment results yield evidence for reduced activation in the precuneus and right inferior parietal lobe after psychological intervention in turn becoming more aligned to the pattern of healthy controls. This might reflect, by taking the models of the functional significance of these structures into account, that after successful exposure treatment, phobia-relevant stimuli no longer trigger an immediate recall of vivid episodic memory contents from a first-person perspective. This suggests a treatment-related reorganization of these memory contents. This re-organization could also be the explanation for trauma-focused EMDR-based treatment approaches aimed at elaborating the worst or “traumatic” dental-related memories proofed effective in DP (Doering et al. 2013).

Our study has several limitations that should be noted and addressed by future investigations. With a total of 34 participants, our overall study sample was quite small, and only one fMRI-measurement was available for healthy controls. We could always check whether the post-treatment changes in the patients were related to the outcome of treatment. However, if this was not the case (as for example in the precuneus and parietal lobe) it was difficult for us to separate treatment effects from pure time and habituation effects, which could also have had an influence on the post-treatment findings due to these design-related limitations. Findings therefore definitely require replication with a larger sample applying a complete 2 (Group) × 2 (Time) design. Our findings and the functional significance of the participating brain structures strongly suggest that pre-treatment confrontation with dental treatment-related stimuli may have triggered self-referential episodic memories of the last or perhaps even the worst dental treatment in phobic study participants which is only based on anecdotal evidence. Finally, it should not go unmentioned that in addition to the discussed activation patterns, we observed differential activity in primary and right premotor cortex areas as well as the supramarginal gyrus which so far can only be poorly classified in the relevant literature which again underlines the importance of a replication study.

To summarize, neural findings are consistent with the idea that, as typical for phobic disorders, DP is associated with a hyperactivation of fear-sensitive brain areas and that attenuated activity in these areas is a function of successful exposure treatment. However, current findings also suggest that DP may also be characterized by disturbed memory retrieval, such that exposure to phobia-related cues leads to an immediate recall of episodic dental-related memories from a first-person perspective. The post-treatment decrease in activation in the relevant brain areas may be a sign of a reorganization of these memory contents as a result of the treatment.

### Supplementary Information

Below is the link to the electronic supplementary material.

Supplementary file1 (DOCX 16 kb)

## Data Availability

Data will be made available on reasonable request.
