# Supplementary material for: More than fear? Brain activation patterns of dental phobic patients before and after an exposure-based treatment
Source: J Neural Transm (Vienna). 2024 Mar 1;131(4):393–404. doi: 10.1007/s00702-024-02754-6 (PMC11016127; doi:10.1007/s00702-024-02754-6)
Supplement: Supplementary file 1 — Supplementary file1 (DOCX 16 kb) [file 702_2024_2754_MOESM1_ESM.docx]

**Supplementary Material**

**Supplementary Table 1**: Pictures and sounds applied in randomized order in the fmri-experiment.

| Applied pictures | | | | Applied sounds | | | |
| --- | --- | --- | --- | --- | --- | --- | --- |
| neutral | | phobia-related | | neutral | | phobia-related | |
| IAPS | Title | IAPS | Title | IADS | Title | IADS | Title |
| 5130 | “Rocks” | 3280 | “DentalExam” | 101 | “Cat” | - | “TurbineBurr” |
| 5500 | “Mushroom” | 9582 | “DentalExam” | 132 | “Chickens” | - | “TurbineBurr” |
| 7000 | “RollingPin” | 9584 | “DentalExam” | 171 | “CountryNight” | - | “TurbineBurr” |
| 7002 | “Towel” | - | “ToothExtraction” | 251 | “NoseBlow” | - | “TurbineBurr” |
| 7004 | “Spoon” | - | “ToothExtraction” | 262 | “Yawn” | - | “TurbineBurr” |
| 7009 | “Mug” | - | “Instruments” | 311 | “Crowd2” | - | “TurbineBurr” |
| 7036 | “Shipyard” | - | “Instruments” | 320 | “Office1” | - | “RoseHead Burr” |
| 7038 | “Shoes” | - | “ExtractedTooth” | 322 | “TypeWriter” | - | “RoseHead Burr” |
| 7040 | “DustPan” | - | “OralCavity Inspection” | 325 | “Traffic” | - | “RoseHead Burr” |
| 7050 | “HairDryer” | - | “DentalMirrorIn-spection” | 358 | “Writing” | - | “RoseHead Burr” |
| 7060 | “TrashCan” | - | “DentalMirrorIn-spection” | 425 | “Train” | - | “RoseHead Burr” |
| 7080 | “Fork” | - | “DentalMirrorIn-spection” | 602 | “Thunderstorm” | - | “UltrasonicTartar Remover” |
| 7090 | “Book” | - | “DentalTreatmentPractice” | 700 | “ToiletFlush” | - | “UltrasonicTartar Remover” |
| 7100 | “FireHydrant” | - | “DentalTreatmentPractice” | 701 | “Fan” | - | “UltrasonicTartar Remover” |
| 7110 | “Hammer” | - | “Burr” | 704 | “TouchTone” | - | “SonicTartar Remover” |
| 7130 | “Truck” | - | “TartarRemoval” | 707 | “Urinating” | - | “SonicTartar Remover” |
| 7140 | “Bus” | - | “Dentist” | 708 | “ClockTick” | - | “SonicTartar Remover” |
| 7160 | “Fabric” | - | “Dentist” | 724 | “Chewing” | - | “SuctionDevice” |
| 7500 | “Building” | - | “InjectionSyringe” | 725 | “SodaFizz” | - | “SuctionDevice” |
| 7550 | “Office” | - | “DentalProbe Tooth” | 726 | “Cork” | - | “SuctionDevice” |

Note: In the case that the stimulus came from the IAPS/IADS, the name of the image corresponds to the respective IAPS/IADS titles. The titles of the other images are self-selected and reflect the image/noise content
